# Temporal and geographic trends in extended-spectrum cephalosporins resistance among *Neisseria gonorrhoeae* isolates worldwide: a systematic review and meta-analysis

**DOI:** 10.1186/s12879-025-11601-2

**Published:** 2025-09-29

**Authors:** Laith B. Alhusseini, Bita Hasani, Firas Nabeeh Jaafar, Masoumeh Beig, Sara Abbasian, Khalil Azizian

**Affiliations:** 1 Department of Ecology, College of Science, Kufa University, Kufa, Najaf, Iraq; 2 Department of Microbiology, Islamic Azad University, Tonekabon, Iran; 3 Microbiology Department, College of Science, Mustansiriyah University, Baghdad, Iraq; 4 Department of Bacteriology, Pasteur Institute of Iran, Tehran, Iran; 5 Student Research Committee, Pasteur Institute of Iran, Tehran, Iran; 6 Laboratory Sciences Research Center, Golestan University of Medical Sciences, Gorgan, Iran; 7 Department of Microbiology, Faculty of Medicine, Kurdistan University of Medical Sciences, Sanandaj, Iran; 8 Zoonoses Research Center, Research Institute for Health Development, Kurdistan University of Medical Sciences, Sanandaj, Iran

**Keywords:** *Neisseria gonorrhoeae*, Antimicrobial resistance, Third-generation extended-spectrum cephalosporin, Systematic review and meta-analysis

## Abstract

The global emergence of antibiotic resistance in *Neisseria gonorrhoeae* (NG) infections poses a critical public health challenge. This study aimed to evaluate global resistance rates to extended-spectrum cephalosporins (ESCs) in *N. gonorrhoeae*, considering factors such as time, geography, antimicrobial susceptibility testing (AST), and resistance interpretation. A systematic review and meta-analysis (from 1988 to 2025) of 252 studies from 71 countries reported a weighted pooled resistance rate (WPR) of ≤ 2.5% for ESCs. Significant temporal variation in ESCs-resistant isolates (*P* < 0.05) underscores the dynamic nature of resistance development. Significantly, there was a difference in penicillin resistance rates between countries/ continents, and AST (*P* < 0.001). These findings emphasize the urgent need for effective antimicrobial stewardship, enhanced contact tracing, and comprehensive monitoring systems to combat antimicrobial resistance in gonococcal infections.

## Introduction

As the second most common bacterial STI (Sexually transmitted infection), gonorrhea continues to be a major public health problem [1, 2]. The World Health Organization (WHO) estimates that in 2020, between the ages of 15 and 49, there were 82.4 million new cases of gonorrhea reported [3]. The WHO estimates that there were 23.4 million cases in the Western Pacific area and 21.8 million cases in the African region [3]. *Neisseria gonorrhoeae* (NG), the causative agent of gonorrhea, has exhibited a significant increase in antimicrobial resistance (AMR) worldwide, particularly in relation to extended-spectrum cephalosporins (ESCs), which are the last available option for first-line empirical therapy in many countries [4–6]. Current guidelines recommend dual therapy with ceftriaxone and azithromycin for gonorrhea, but rising resistance to both agents threatens treatment efficacy. Alternative options such as cefixime, gentamicin, or spectinomycin are used in some settings, though with limitations. Novel agents like zoliflodacin are in development, highlighting the need for ongoing resistance surveillance to guide treatment and preserve existing therapies [4–6]. Additionally, there have been reports of decreased susceptibility to ceftriaxone and other antibiotic therapies in various parts of the world [4, 6–8]. This might result in an increase in serious consequences such ectopic pregnancy, infertility, and increased HIV transmission [4, 6–8]. For the purpose of managing gonorrhea, it is crucial to guarantee the availability of an efficient, economical, and accessible antimicrobial therapy. Despite growing concerns, recent global meta-analyses on ESCs resistance are lacking, particularly comprehensive assessments with regional insights. This study addresses that gap by providing updated, geographically stratified estimates of resistance trends to inform treatment guidelines and surveillance efforts. Therefore, it is imperative to ensure the availability of effective, accessible, and affordable antimicrobial treatment for the management of gonorrhea. The aim of this comprehensive examination and synthesis of studies is to evaluate the combined resistance rate of NG strains to ceftriaxone, cefixime, and cefotaxime across the globe.

## Methods

Following the recommendations given in the Preferred Reporting Items for Systematic Reviews and Meta Analyses (PRISMA) [9], this review was carried out.

### Search strategy and study selection

A methodical approach was taken in Looking for pertinent papers and choosing research. Up until July 2025, we carried out thorough searches in the databases PubMed, Scopus, and Embase. The searches were conducted using specific keywords related to: (“*Neisseria gonorrhoeae*” OR “Gonorrhoea” OR “Gonococcus”) AND (“antimicrobial resistance” OR “antibiotic resistance” OR “beta-lactam” OR “ceftriaxone” OR “cefixime” OR “cefotaxime”). No limitations were applied during the database searches. However, the study’s abstract has to be accessible in English in order to be included in our analysis. The researchers that worked on the study devised and carried out the search plan. To find any other articles that would be relevant, the reference lists of all pertinent papers were also examined. After merging the records found in the database searches, EndNote X8 (Thomson Reuters, NY) was used to eliminate duplicates. After evaluating the search results at random, one of our team members made sure that no pertinent studies had been missed. The writers carried out each of these actions, and they had a conversation to settle any disputes about the articles’ selection.

### Inclusion and exclusion criteria

The analyses incorporated in this study adhered to a set of specific criteria. Initial requirements necessitated that the studies be original investigations focused on AMR in human clinical isolates of NG. Furthermore, these studies were required to have undergone peer review and be published in the English Language between January 1988 and August 2025. Additionally, it was imperative that the total number of NG isolates tested be explicitly stated. Finally, the studies were expected to provide a comprehensive description of the resistance testing methods employed, encompassing both minimum inhibitory concentration (MIC)-based techniques, disk diffusion methods, and a combination of the two. Finally, the investigations were required to provide the AMR rate in NG isolates based on the criteria established by the Clinical and Laboratory Standards Institute (CLSI), the European Committee on Antimicrobial Susceptibility Testing (EUCAST), and/or the World Health Organization (WHO) WPR Resistance Surveillance Programme guidelines [10–13]. The CLSI identified reduced susceptibilities to extended-spectrum cephalosporins (ESCs) as a cefixime or ceftriaxone MIC of ≥ 0.25 mg/L, and a cefotaxime MIC of ≥ 0.5 mg/L. The EUCAST defined clinical resistance breakpoints to the ESCs as a MIC of > 0.125 mg/L. Studies that included duplicated data or overlapping articles, as well as those that did not involve clinical NG isolates, animal research, reviews, meta-analyses, systematic reviews, conference abstracts, or articles lacking full text, were excluded from the analysis. Furthermore, studies that did not present or report resistance rates were also excluded.

### Data extraction

The information extracted from each study comprised of various elements, including the first author, the year of the study, publication year, continent, country, number of clinical NG isolates, number of resistant NG isolates, the method employed for antibiotic susceptibility testing (AST), and the interpretation of resistance based on CLSI, EUCAST, and WHO criteria. The process of data extraction was carried out by an independent examiner and subsequently verified by another researcher.

### Quality assessment

To assess the quality of the studies included, an adapted version of the Newcastle-Ottawa assessment scale for cross-sectional studies [14] was utilized. Each study was assigned a score ranging from 0 to 8 points, with a score of 6 or higher indicating a high quality study and a score of 5 or lower indicating a low quality study. In cases where there was disagreement, the assessment was reviewed by a designated adjudicator.

### Publication bias

Publication bias, also known as the small study effect, was analysed using Egger’s linear regression test.

### Statistical analysis

The meta-analysis encompassed cross-sectional studies that furnished original data on antibiotic susceptibility in clinical NG isolates. The analysis was executed utilizing a random-effects model through the utilization of Stata/SE software. The computation of the weighted pooled resistance (WPR) was derived from the Freeman-Tukey double-arcsine transformation. The evaluation of heterogeneity across studies was carried out employing forest plots and the I^2^ statistic, whereby I^2^ values of 25%, 50%, and 75% indicated low, medium, and high heterogeneity, respectively. The DerSimonian and Laird random effects models were implemented. Meta-regression models were employed to investigate the variation in antibiotic resistance over time. Subgroup analyses were performed on the basis of publication Year, geographic areas, AST, and interpretation of resistance. All statistical interpretations were reported along with a 95% confidence interval [15].

### Study outcomes

The primary outcome was the proportion of clinical NG isolates resistant to penicillin and ESCs (ceftriaxone, cefixime, and cefotaxime). To better study the data, we looked at different groups based on when the publication was released (1988–2013, 2014–2018, and 2019–2025), different regions (continents), AST, and different ways of interpreting the results according to the CLSI, EUCAST, and the WHO. The main outcome they were looking at was how often bacteria became resistant to ceftriaxone, cefixime, and cefotaxime when treating patients with clinical NG. A subgroup analysis was performed; first, we looked at different time periods, then at different regions (continents), AST, and how resistance was interpreted (CLSI, EUCAST, and WHO).

## Results

### Systematic literature search

**Fig. 1:**
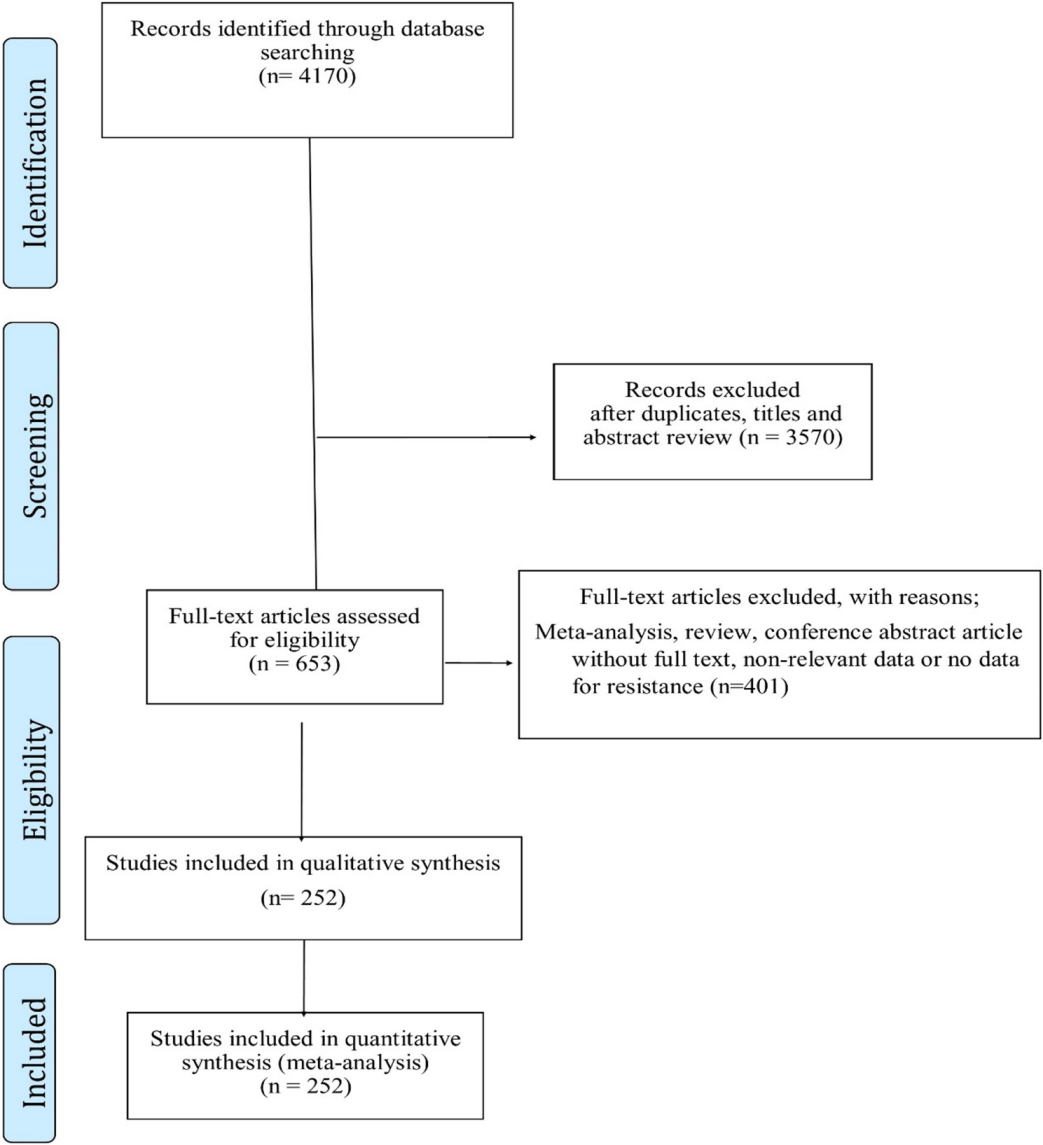
Flow chart of study selection

We found 4170 articles during our thorough research. After going through the title and Summary, 3517 articles were Left out because they were not related or duplicated. The remaining 653 articles were subjected to full-text review (Fig. 1). Among these, 401 articles were excluded for various reasons, Such as duplicate data, overlapping articles, absence of clinical NG isolates, inclusion of reviews, meta-analyses, systematic reviews, conference abstracts, or articles without full text, and lack of reported resistance rates. Finally, a total of 252 studies published between 1988 and 2025 were included in the analysis [8, 16–267].

### Characteristics of included studies

**Fig. 2:**
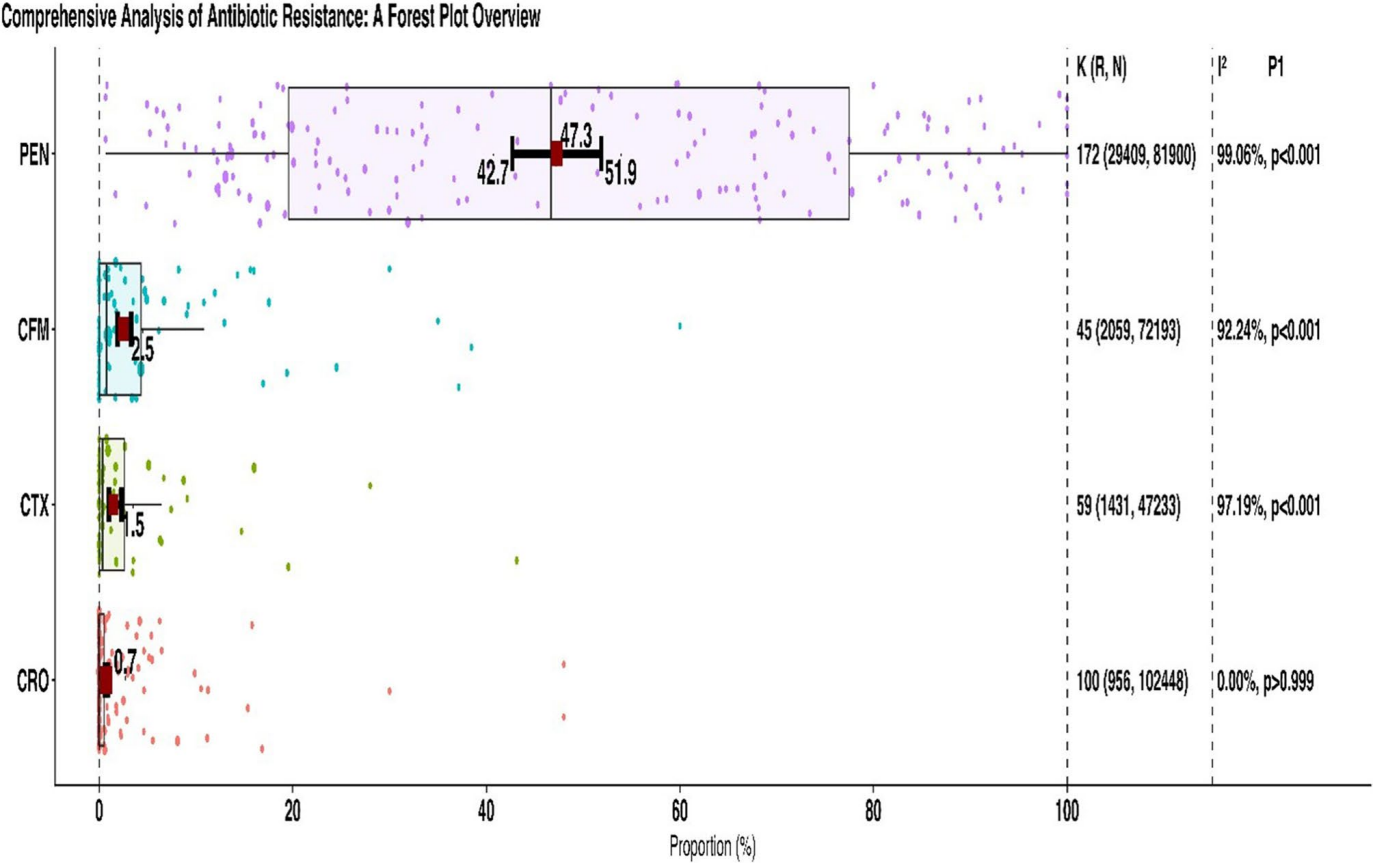
Weighted pooled resistance rates for each antimicrobial in the study

**Fig. 3:**
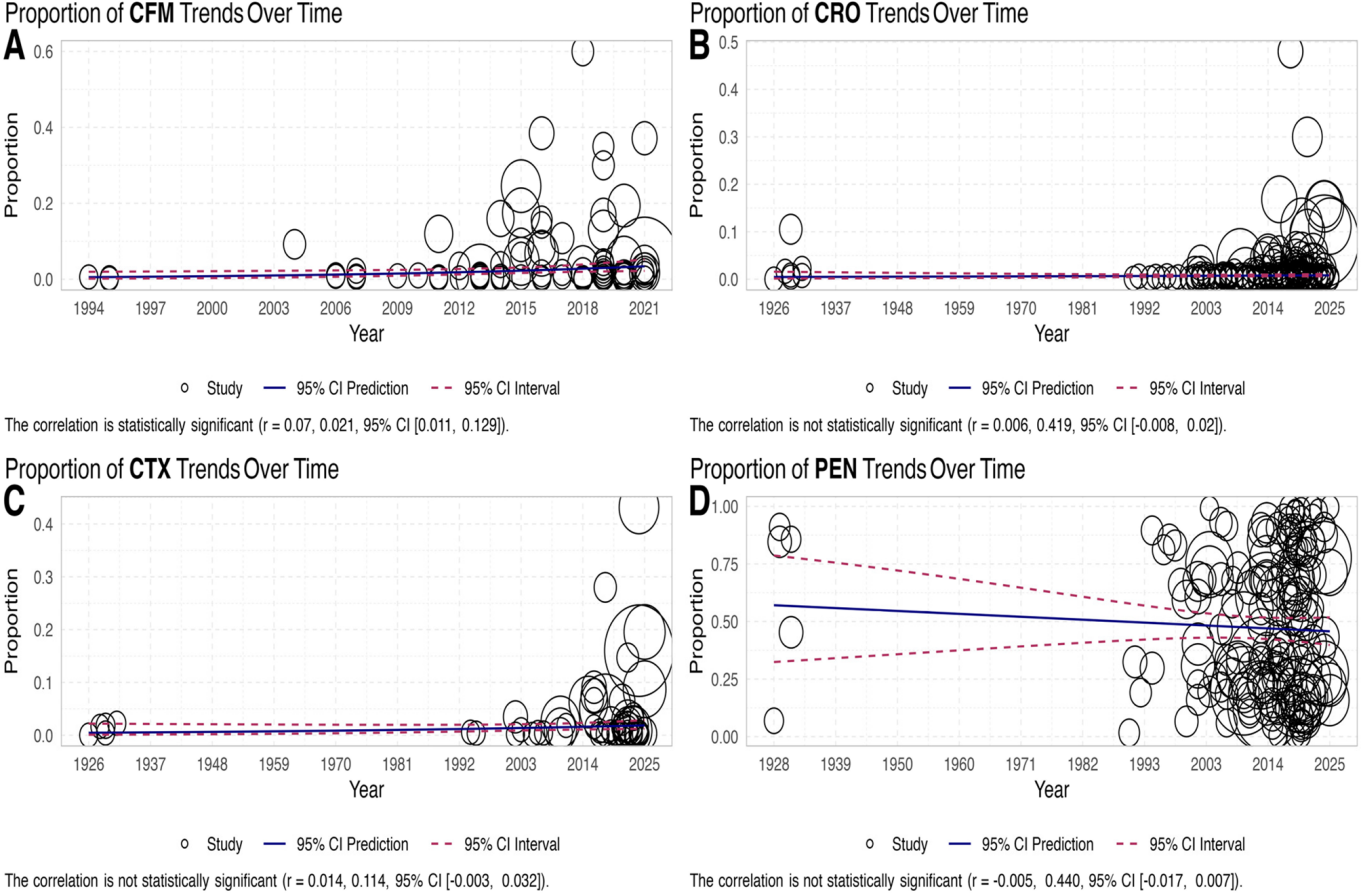
Meta-regression analysis in scatter plot of trend of proportion of antibiotic-resistant isolates during years

The reports came from 71 countries (Canada, United States, Indonesia, Tanzania, China, Finland, Gambia, Liberia, Bangladesh, India, South Africa, Brazil, Cuba, Israel, Nigeria, Japan, South Korea, Turkey, Germany, Russia, Armenia, Italy, Denmark, Mozambique, Malawi, Belarus, Kenya, Norway, Guinea-Bissau, Vietnam, Morocco, France, Hungary, Slovenia, Estonia, Poland, Uganda, Zimbabwe, Spain, Thailand, Pakistan, Iran, Taiwan, Argentina, Philippines, Netherlands, New Zealand, Cameroon, Laos, Bhutan, Ethiopia, Ireland, Ukraine, Côte d’Ivoire, Ghana, Portugal, United Kingdom, Jamaica, Kyrgyzstan, Qatar, Peru, Sweden, Austria, Zambia, Panama, Puerto Rico, Australia, Greece, Cambodia, Switzerland, Cyprus). The proportion of resistant isolates to these antibiotics are depicted in Fig. 2; Table 1. The Table 1 shows the percentage of resistance to each antibiotic and additional information based on the year of publication, location, methods used in AST, and how resistance is interpreted. Figure 3 shows a meta-regression of the temporal trend in the proportion of antibioticresistant isolates. The information below shows the current rates of resistance to penicillin, ceftriaxone, cefixime, and cefotaxime.

**Table 1:** Subgroup analysis for penicillin, ceftriaxone, cefixime, and cefotaxime resistance rates

Antibiotic	Category	Subgroup	K (*n*, *N*)	Proportion 95%CI(LCI, HCI)	I²	*P*. value
Cefixime	Overall		92 (2059, 72193)	0.025 (0.019, 0.033)	92.24%	
	Countries	United States	4 (230, 26149)	0.009 (0.008, 0.010)	0.00%	*p* < 0.001
		Indonesia	2 (0, 121)	0.009 (0.001, 0.061)	0.00%	
		Tanzania	1 (0, 130)	0.004 (0.000, 0.058)	0.00%	
		Japan	6 (296, 1972)	0.098 (0.054, 0.171)	89.59%	
		Germany	4 (9, 1249)	0.007 (0.002, 0.026)	42.21%	
		China	6 (206, 2020)	0.121 (0.059, 0.232)	95.68%	
		India	3 (7, 204)	0.031 (0.001, 0.532)	88.19%	
		Russia	1 (0, 76)	0.006 (0.000, 0.095)	0.00%	
		Armenia	1 (0, 101)	0.005 (0.000, 0.073)	0.00%	
		Mozambique	1 (0, 55)	0.009 (0.001, 0.127)	0.00%	
		Malawi	1 (1, 100)	0.010 (0.001, 0.068)	0.00%	
		Belarus	3 (15, 795)	0.015 (0.004, 0.047)	42.60%	
		Canada	2 (2, 261)	0.012 (0.003, 0.048)	12.24%	
		Italy	3 (63, 425)	0.146 (0.045, 0.383)	93.47%	
		Norway	2 (14, 1072)	0.018 (0.005, 0.058)	76.49%	
		Kenya	2 (0, 176)	0.008 (0.001, 0.057)	0.00%	
		Vietnam	2 (2, 216)	0.009 (0.002, 0.036)	0.00%	
		Brazil	2 (0, 317)	0.003 (0.000, 0.023)	0.00%	
		Morocco	1 (0, 72)	0.007 (0.000, 0.100)	0.00%	
		Hungary	3 (6, 826)	0.012 (0.003, 0.053)	69.40%	
		Slovenia	1 (31, 194)	0.160 (0.115, 0.218)	0.00%	
		Poland	1 (0, 228)	0.002 (0.000, 0.034)	0.00%	
		Uganda	2 (1, 164)	0.011 (0.002, 0.053)	0.00%	
		Zimbabwe	1 (0, 66)	0.007 (0.000, 0.108)	0.00%	
		Spain	8 (191, 5939)	0.042 (0.023, 0.075)	90.24%	
		South Korea	1 (19, 210)	0.090 (0.058, 0.137)	0.00%	
		Bangladesh	2 (18, 46)	0.345 (0.058, 0.819)	88.49%	
		Iran	1 (5, 32)	0.156 (0.067, 0.325)	0.00%	
		Thailand	2 (0, 3432)	0.001 (0.000, 0.005)	29.61%	
		Pakistan	1 (0, 100)	0.005 (0.000, 0.074)	0.00%	
		Taiwan	4 (73, 1649)	0.008 (0.001, 0.076)	80.68%	
		Philippines	2 (0, 107)	0.011 (0.002, 0.077)	0.00%	
		Ireland	1 (6, 609)	0.010 (0.004, 0.022)	0.00%	
		Ukraine	2 (0, 300)	0.003 (0.000, 0.023)	0.00%	
		Côte d’Ivoire	1 (0, 212)	0.002 (0.000, 0.036)	0.00%	
		Denmark	1 (0, 191)	0.003 (0.000, 0.040)	0.00%	
		United Kingdom	1 (8, 1277)	0.006 (0.003, 0.012)	0.00%	
		Kyrgyzstan	1 (2, 156)	0.013 (0.003, 0.050)	0.00%	
		Cyprus	1 (1, 45)	0.022 (0.003, 0.142)	0.00%	
	Continents	Americas	8 (232, 26727)	0.009 (0.008, 0.010)	0.00%	*p* < 0.001
		Asia	35 (629, 10411)	0.051 (0.034, 0.075)	92.03%	
		Africa	10 (2, 975)	0.008 (0.003, 0.017)	0.00%	
		Europe	30 (335, 11904)	0.024 (0.015, 0.038)	91.90%	
		NA	9 (861, 22176)	0.031 (0.020, 0.047)	91.54%	
	AST method	MIC base	80 (1946, 68914)	0.022 (0.016, 0.030)	96.40%	*p* = 0.065
		Disc diffusion	11 (103, 2321)	0.051 (0.018, 0.139)	89.60%	
		Mixed	1 (10, 958)	0.010 (0.006, 0.019)	0.00%	
	AST guidelines	CLSI	43 (697, 38353)	0.017 (0.010, 0.031)	97.12%	*p* = 0.760
		WHO	4 (49, 640)	0.017 (0.002, 0.147)	79.85%	
		EUCAST	45 (1313, 33200)	0.030 (0.021, 0.041)	94.63%	
	Year group	1988–2013	26 (282, 28739)	0.009 (0.005, 0.019)	89.85%	*p* = 0.002
		2014–2018	33 (665, 14017)	0.037 (0.024, 0.057)	94.65%	
		2019–2025	33 (1112, 29437)	0.033 (0.022, 0.048)	94.56%	
CRO	Overall		201 (956, 102448)	0.007 (0.006, 0.009)	0.00%	
	Countries	Canada	6 (0, 5994)	0.001 (0.000, 0.004)	6.04%	*p* < 0.001
		United States	7 (0, 1097)	0.005 (0.002, 0.013)	0.00%	
		Indonesia	2 (0, 121)	0.009 (0.001, 0.061)	0.00%	
		China	33 (643, 31553)	0.020 (0.014, 0.031)	94.01%	
		Finland	1 (0, 337)	0.001 (0.000, 0.023)	0.00%	
		Liberia	1 (0, 100)	0.005 (0.000, 0.074)	0.00%	
		South Africa	7 (0, 2115)	0.003 (0.001, 0.009)	0.00%	
		Brazil	6 (1, 4666)	0.001 (0.000, 0.004)	0.00%	
		Bangladesh	4 (13, 2123)	0.013 (0.000, 0.428)	94.57%	
		India	6 (0, 400)	0.009 (0.003, 0.027)	0.00%	
		Israel	3 (0, 1711)	0.001 (0.000, 0.007)	0.00%	
		Cuba	1 (0, 91)	0.005 (0.000, 0.081)	0.00%	
		Japan	8 (0, 1679)	0.003 (0.001, 0.008)	0.00%	
		Turkey	1 (0, 78)	0.006 (0.000, 0.093)	0.00%	
		Germany	5 (12, 2499)	0.005 (0.002, 0.013)	25.53%	
		Russia	4 (1, 1942)	0.003 (0.001, 0.013)	20.51%	
		Armenia	1 (0, 101)	0.005 (0.000, 0.073)	0.00%	
		Italy	3 (0, 686)	0.003 (0.001, 0.013)	0.00%	
		Denmark	3 (0, 549)	0.003 (0.001, 0.016)	0.00%	
		Mozambique	1 (0, 55)	0.009 (0.001, 0.127)	0.00%	
		Malawi	2 (0, 283)	0.004 (0.001, 0.026)	0.00%	
		Guinea-Bissau	1 (0, 31)	0.016 (0.001, 0.206)	0.00%	
		Norway	2 (2, 1072)	0.004 (0.000, 0.105)	79.89%	
		Kenya	4 (1, 323)	0.009 (0.003, 0.032)	0.00%	
		Vietnam	2 (10, 216)	0.046 (0.025, 0.084)	0.00%	
		Morocco	1 (0, 72)	0.007 (0.000, 0.100)	0.00%	
		Hungary	3 (0, 826)	0.003 (0.001, 0.014)	0.00%	
		Slovenia	1 (10, 194)	0.052 (0.028, 0.093)	0.00%	
		Estonia	1 (1, 44)	0.023 (0.003, 0.144)	0.00%	
		Poland	3 (0, 319)	0.007 (0.001, 0.033)	0.00%	
		Uganda	2 (0, 164)	0.010 (0.001, 0.079)	15.75%	
		Zimbabwe	1 (0, 66)	0.007 (0.000, 0.108)	0.00%	
		Spain	12 (74, 8641)	0.012 (0.007, 0.020)	64.05%	
		Belarus	2 (0, 715)	0.002 (0.000, 0.011)	0.00%	
		South Korea	2 (7, 244)	0.029 (0.014, 0.059)	0.00%	
		Iran	2 (6, 70)	0.088 (0.040, 0.183)	0.00%	
		Pakistan	1 (0, 100)	0.005 (0.000, 0.074)	0.00%	
		Taiwan	6 (63, 2449)	0.012 (0.003, 0.054)	94.72%	
		Cameroon	2 (9, 588)	0.016 (0.008, 0.030)	0.00%	
		Laos	1 (0, 158)	0.003 (0.000, 0.048)	0.00%	
		Bhutan	1 (0, 381)	0.001 (0.000, 0.021)	0.00%	
		Thailand	4 (0, 3983)	0.001 (0.000, 0.006)	23.72%	
		Ethiopia	6 (12, 758)	0.018 (0.001, 0.217)	89.14%	
		Ireland	1 (0, 609)	0.001 (0.000, 0.013)	0.00%	
		Ukraine	2 (0, 300)	0.003 (0.000, 0.023)	0.00%	
		Côte d’Ivoire	1 (0, 212)	0.002 (0.000, 0.036)	0.00%	
		Argentina	1 (3, 158)	0.019 (0.006, 0.057)	0.00%	
		Netherlands	1 (0, 80)	0.006 (0.000, 0.091)	0.00%	
		Ghana	2 (0, 100)	0.010 (0.001, 0.067)	0.00%	
		United Kingdom	1 (0, 1277)	0.000 (0.000, 0.006)	0.00%	
		Kyrgyzstan	1 (0, 156)	0.003 (0.000, 0.049)	0.00%	
		Jamaica	1 (0, 91)	0.005 (0.000, 0.081)	0.00%	
		Australia	1 (0, 1176)	0.000 (0.000, 0.007)	0.00%	
		Qatar	1 (0, 433)	0.001 (0.000, 0.018)	0.00%	
		Sweden	2 (4, 7980)	0.001 (0.000, 0.001)	0.00%	
		New Zealand	1 (0, 314)	0.002 (0.000, 0.025)	0.00%	
		Cyprus	1 (0, 45)	0.011 (0.001, 0.151)	0.00%	
		Puerto Rico	1 (0, 50)	0.010 (0.001, 0.138)	0.00%	
		Austria	2 (33, 1750)	0.007 (0.000, 0.128)	78.85%	
		Zambia	1 (0, 122)	0.004 (0.000, 0.062)	0.00%	
		Cambodia	1 (47, 306)	0.154 (0.117, 0.198)	0.00%	
		Panama	1 (0, 30)	0.016 (0.001, 0.211)	0.00%	
		Tanzania	1 (1, 164)	0.006 (0.001, 0.042)	0.00%	
		Switzerland	1 (0, 15)	0.031 (0.002, 0.350)	0.00%	
	Continents	Americas	24 (4, 12177)	0.003 (0.002, 0.006)	22.31%	*p* < 0.001
		Asia	81 (789, 46307)	0.014 (0.010, 0.019)	91.08%	
		Europe	49 (137, 28558)	0.006 (0.004, 0.009)	70.63%	
		Africa	33 (23, 5153)	0.008 (0.004, 0.017)	75.54%	
		NA	12 (3, 8763)	0.002 (0.001, 0.005)	32.32%	
		Oceania	2 (0, 1490)	0.001 (0.000, 0.006)	0.00%	
	AST method	MIC base	174 (861, 96293)	0.007 (0.005, 0.009)	88.93%	*p* = 0.033
		Mixed	8 (41, 2682)	0.007 (0.001, 0.035)	91.16%	
		Disc diffusion	18 (54, 3452)	0.017 (0.006, 0.049)	89.43%	
	AST guidelines	CLSI	104 (239, 42399)	0.006 (0.004, 0.010)	86.34%	*p* = 0.251
		WHO	13 (75, 11914)	0.005 (0.002, 0.013)	92.82%	
		EUCAST	82 (642, 46764)	0.009 (0.007, 0.013)	90.22%	
		EUCAST/CLSI	2 (0, 1371)	0.001 (0.000, 0.005)	0.00%	
	Year group	1988–2013	72 (174, 44113)	0.005 (0.003, 0.007)	75.08%	*p* = 0.010
		2014–2018	50 (129, 20693)	0.007 (0.004, 0.012)	87.81%	
		2019–2025	79 (653, 37642)	0.011 (0.008, 0.016)	90.24%	
CTX	Overall		59 (1431, 47233)	0.015 (0.010, 0.023)	97.19%	NA
	Countries	Indonesia	2 (0, 353)	0.003 (0.000, 0.023)	0.00%	*p* < 0.001
		Tanzania	2 (2, 294)	0.010 (0.003, 0.033)	0.00%	
		China	5 (563, 3812)	0.094 (0.055, 0.157)	90.64%	
		Spain	5 (98, 3547)	0.033 (0.015, 0.069)	85.46%	
		Taiwan	3 (15, 632)	0.010 (0.001, 0.145)	82.69%	
		Netherlands	2 (178, 3561)	0.028 (0.004, 0.165)	56.76%	
		Ethiopia	3 (7, 343)	0.029 (0.001, 0.488)	87.41%	
		Ireland	1 (13, 609)	0.021 (0.012, 0.036)	0.00%	
		Denmark	1 (1, 191)	0.005 (0.001, 0.036)	0.00%	
		Portugal	1 (1, 425)	0.002 (0.000, 0.017)	0.00%	
		Poland	1 (0, 26)	0.019 (0.001, 0.236)	0.00%	
		Jamaica	1 (0, 91)	0.005 (0.000, 0.081)	0.00%	
		Italy	1 (26, 1525)	0.017 (0.012, 0.025)	0.00%	
		Australia	1 (74, 1176)	0.063 (0.050, 0.078)	0.00%	
		Israel	1 (105, 1205)	0.087 (0.072, 0.104)	0.00%	
		United Kingdom	1 (22, 2904)	0.008 (0.005, 0.011)	0.00%	
		Brazil	4 (13, 4893)	0.003 (0.002, 0.005)	0.00%	
		Sweden	2 (83, 7980)	0.012 (0.006, 0.023)	85.82%	
		South Africa	4 (1, 867)	0.005 (0.001, 0.016)	0.00%	
		Japan	1 (3, 85)	0.035 (0.011, 0.104)	0.00%	
		United States	1 (0, 29)	0.017 (0.001, 0.217)	0.00%	
		New Zealand	1 (0, 314)	0.002 (0.000, 0.025)	0.00%	
		Thailand	2 (0, 741)	0.002 (0.000, 0.013)	0.00%	
		Austria	2 (42, 1750)	0.023 (0.015, 0.036)	31.43%	
		South Korea	1 (5, 34)	0.147 (0.063, 0.308)	0.00%	
		Cambodia	1 (132, 306)	0.431 (0.377, 0.487)	0.00%	
		Panama	1 (0, 30)	0.016 (0.001, 0.211)	0.00%	
		Ghana	1 (1, 56)	0.018 (0.003, 0.116)	0.00%	
		Canada	1 (0, 2394)	0.000 (0.000, 0.003)	0.00%	
		Malawi	1 (0, 183)	0.003 (0.000, 0.042)	0.00%	
		Switzerland	1 (1, 15)	0.067 (0.009, 0.352)	0.00%	
	Continents	Asia	16 (823, 7168)	0.054 (0.032, 0.089)	95.13%	*p* < 0.001
		Africa	11 (11, 1743)	0.010 (0.002, 0.043)	81.90%	
		Europe	18 (465, 22533)	0.020 (0.013, 0.030)	92.86%	
		Americas	8 (13, 7437)	0.003 (0.002, 0.005)	0.00%	
		Oceania	2 (74, 1490)	0.013 (0.000, 0.337)	85.60%	
		NA	4 (45, 6862)	0.005 (0.002, 0.015)	78.67%	
	AST method	MIC base	52 (1408, 45980)	0.015 (0.010, 0.023)	97.48%	*p* = 0.857
		Mixed	3 (16, 707)	0.012 (0.001, 0.145)	85.88%	
		Disc diffusion	3 (7, 525)	0.014 (0.000, 0.465)	91.20%	
	AST guidelines	CLSI	24 (43, 6603)	0.010 (0.005, 0.020)	78.58%	*p* = 0.035
		WHO	1 (0, 130)	0.004 (0.000, 0.058)	0.00%	
		EUCAST	32 (1387, 39129)	0.022 (0.013, 0.037)	98.36%	
		EUCAST/CLSI	2 (1, 1371)	0.001 (0.000, 0.006)	0.00%	
	Year group	1988–2013	17 (126, 13376)	0.008 (0.005, 0.015)	78.16%	*p* = 0.035
		2014–2018	10 (286, 6051)	0.058 (0.038, 0.089)	80.68%	
		2019–2025	32 (1019, 27806)	0.015 (0.008, 0.026)	98.00%	
PEN	Overall		172 (29409, 81900)	0.473 (0.427, 0.519)	99.06%	
	Countries	Canada	5 (1868, 12300)	0.168 (0.113, 0.242)	97.87%	*p* < 0.001
		United States	7 (156, 523)	0.250 (0.131, 0.425)	92.28%	
		Indonesia	3 (291, 388)	0.867 (0.623, 0.963)	90.43%	
		China	23 (11009, 14905)	0.765 (0.715, 0.808)	97.47%	
		Gambia	1 (95, 111)	0.856 (0.778, 0.910)	0.00%	
		Liberia	1 (83, 100)	0.830 (0.743, 0.892)	0.00%	
		Bangladesh	5 (1058, 2217)	0.525 (0.420, 0.629)	87.53%	
		India	9 (300, 680)	0.498 (0.290, 0.707)	93.40%	
		Cuba	2 (135, 211)	0.640 (0.566, 0.708)	16.17%	
		Israel	3 (285, 1711)	0.169 (0.148, 0.192)	19.90%	
		Nigeria	1 (57, 57)	0.991 (0.877, 0.999)	0.00%	
		Japan	7 (363, 1628)	0.213 (0.141, 0.309)	93.20%	
		South Korea	3 (633, 1061)	0.445 (0.104, 0.846)	98.79%	
		Turkey	1 (20, 78)	0.256 (0.172, 0.364)	0.00%	
		Germany	4 (279, 1249)	0.207 (0.156, 0.269)	80.31%	
		Russia	4 (91, 826)	0.107 (0.062, 0.180)	77.27%	
		Armenia	1 (6, 101)	0.059 (0.027, 0.126)	0.00%	
		Italy	1 (83, 326)	0.255 (0.210, 0.305)	0.00%	
		Denmark	3 (297, 549)	0.389 (0.032, 0.923)	99.02%	
		Malawi	1 (19, 100)	0.190 (0.125, 0.279)	0.00%	
		Belarus	3 (63, 795)	0.080 (0.063, 0.101)	0.00%	
		Kenya	4 (227, 274)	0.907 (0.683, 0.978)	89.19%	
		Guinea-Bissau	1 (21, 31)	0.677 (0.497, 0.817)	0.00%	
		Vietnam	3 (347, 624)	0.525 (0.433, 0.615)	76.10%	
		Brazil	6 (710, 2257)	0.366 (0.267, 0.477)	95.65%	
		Morocco	1 (40, 72)	0.556 (0.440, 0.666)	0.00%	
		France	1 (1003, 7689)	0.130 (0.123, 0.138)	0.00%	
		Hungary	2 (497, 634)	0.821 (0.684, 0.907)	69.07%	
		Slovenia	1 (72, 194)	0.371 (0.306, 0.441)	0.00%	
		Poland	3 (79, 319)	0.255 (0.202, 0.318)	18.18%	
		Uganda	2 (114, 164)	0.700 (0.600, 0.783)	10.26%	
		Spain	12 (1983, 9738)	0.221 (0.181, 0.267)	93.19%	
		Iran	1 (22, 32)	0.688 (0.510, 0.823)	0.00%	
		Pakistan	1 (43, 100)	0.430 (0.337, 0.528)	0.00%	
		Taiwan	4 (1197, 1722)	0.798 (0.622, 0.904)	97.15%	
		Argentina	1 (42, 44)	0.955 (0.836, 0.989)	0.00%	
		Cameroon	1 (185, 198)	0.934 (0.890, 0.961)	0.00%	
		Laos	1 (142, 158)	0.899 (0.841, 0.937)	0.00%	
		Bhutan	1 (378, 381)	0.992 (0.976, 0.997)	0.00%	
		New Zealand	2 (75, 712)	0.103 (0.070, 0.150)	66.56%	
		Ethiopia	6 (536, 758)	0.813 (0.610, 0.923)	94.60%	
		Ukraine	2 (2, 300)	0.007 (0.002, 0.026)	0.00%	
		Côte d’Ivoire	1 (146, 212)	0.689 (0.623, 0.747)	0.00%	
		Ghana	2 (91, 100)	0.910 (0.836, 0.953)	0.00%	
		Portugal	2 (242, 2094)	0.083 (0.031, 0.207)	95.23%	
		Norway	1 (287, 958)	0.300 (0.271, 0.329)	0.00%	
		United Kingdom	1 (212, 1277)	0.166 (0.147, 0.187)	0.00%	
		Philippines	1 (18, 21)	0.857 (0.639, 0.953)	0.00%	
		Kyrgyzstan	1 (61, 156)	0.391 (0.318, 0.470)	0.00%	
		Qatar	1 (133, 433)	0.307 (0.265, 0.352)	0.00%	
		Thailand	2 (198, 216)	0.962 (0.438, 0.999)	83.88%	
		Peru	1 (67, 165)	0.406 (0.334, 0.483)	0.00%	
		South Africa	2 (47, 85)	0.673 (0.229, 0.934)	88.47%	
		Austria	3 (644, 3018)	0.200 (0.126, 0.304)	97.13%	
		Puerto Rico	1 (19, 50)	0.380 (0.257, 0.520)	0.00%	
		Greece	1 (482, 1756)	0.274 (0.254, 0.296)	0.00%	
		Zambia	1 (104, 122)	0.852 (0.778, 0.905)	0.00%	
		Panama	1 (11, 30)	0.367 (0.216, 0.549)	0.00%	
		Tanzania	1 (115, 164)	0.701 (0.627, 0.766)	0.00%	
	Continents	Americas	24 (3008, 15580)	0.325 (0.258, 0.401)	97.73%	*p* < 0.001
		Asia	71 (16504, 26612)	0.610 (0.551, 0.666)	98.45%	
		Africa	26 (1880, 2548)	0.793 (0.720, 0.851)	92.50%	
		Europe	43 (6104, 30445)	0.210 (0.175, 0.250)	97.90%	
		NA	7 (1838, 6003)	0.403 (0.270, 0.553)	97.86%	
		Oceania	2 (75, 712)	0.103 (0.070, 0.150)	66.56%	
	AST method	MIC base	146 (26459, 76013)	0.435 (0.386, 0.484)	99.14%	*p* < 0.001
		Disc diffusion	21 (2229, 3889)	0.737 (0.602, 0.839)	97.57%	
		Mixed	5 (703, 1977)	0.462 (0.213, 0.732)	98.75%	
	AST guidelines	CLSI	102 (16084, 43063)	0.524 (0.467, 0.579)	98.83%	*p* < 0.001
		WHO	11 (3660, 4800)	0.711 (0.570, 0.821)	98.43%	
		EUCAST	60 (9665, 34037)	0.338 (0.270, 0.414)	99.14%	
	Year group	1988–2013	62 (9323, 29005)	0.446 (0.380, 0.514)	98.75%	*p* = 0.575
		2014–2018	45 (9239, 22302)	0.512 (0.407, 0.616)	99.26%	
		2019–2025	66 (10847, 30593)	0.471 (0.393, 0.551)	99.09%	

*CI* Confidence interval, *AST* Antimicrobial Susceptibility Testing, *CLSI* Clinical and Laboratory Standards Institute, *EUCAST* European Committee on Antimicrobial Susceptibility Testing, *WHO* World Health Organization, *MIC* Minimal inhibitory concentration,* n* number of events (drug resistance), *N* Total number of isolates from the included studies

### Penicillin resistance

**Fig. 4:**
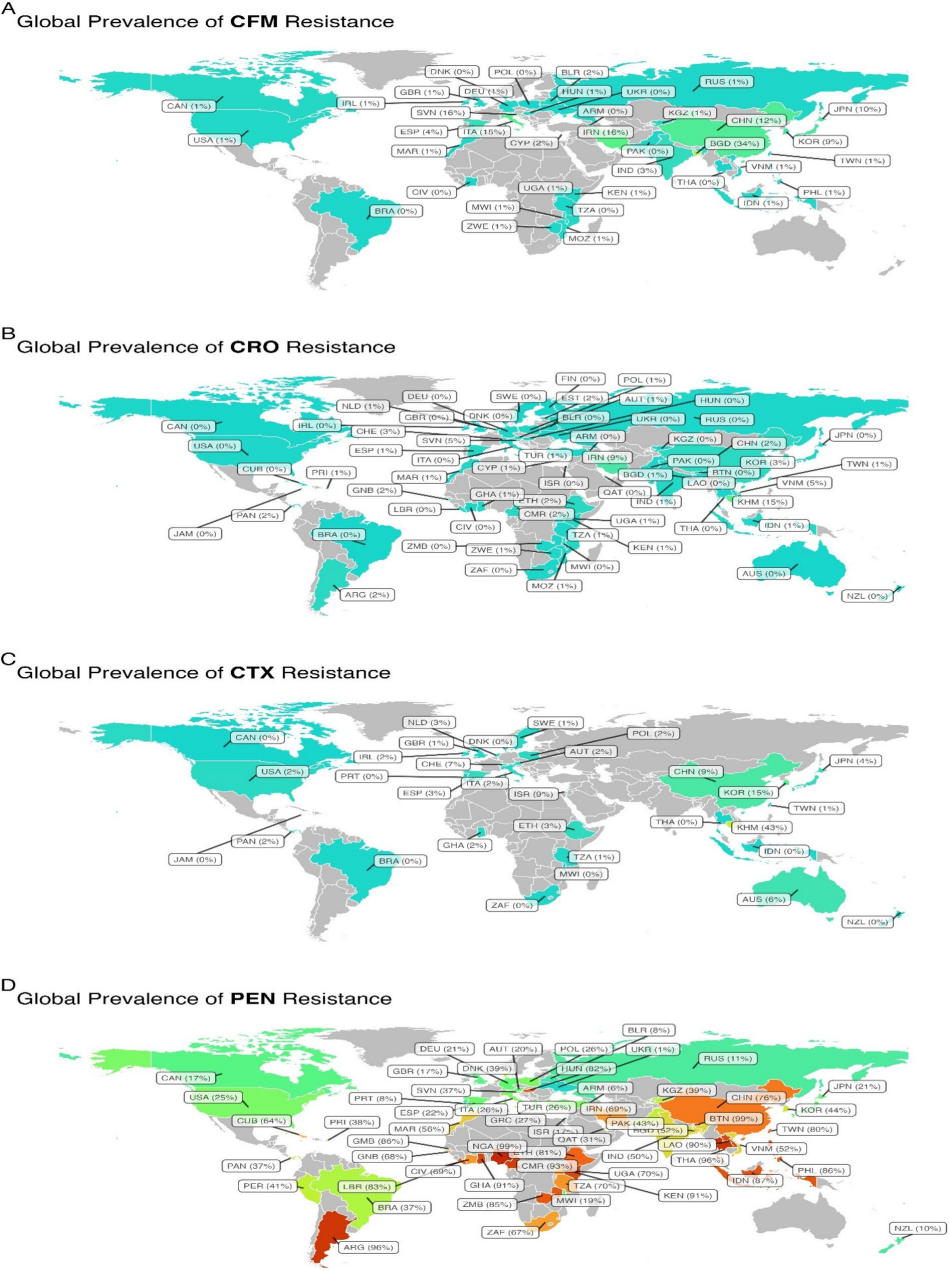
Global map of reported weighted pooled resistance rates for (**A**) cefixime, (**B**) ceftriaxone (**C**) cefotaxime and (**D**) penicillin in the study

The determination of Susceptibility to penicillin was carried out in a total of 173 studies, encompassing 81,900 isolates of NG. The WPR was found to be 47.3% (95% CI 42.7%−51.9%), although there was noticeable heterogeneity (I^2^ = 99.06%, *P* < 0.001) observed among the included articles (Table 1; Fig. 2). Additionally, we found no strong evidence of publication bias (according to the Egger rank correlation test with a p-value of < 0.001). To better understand the patterns of penicillin resistance in recent Years, researchers Looked at three different time periods: 1988–2013, 2014–2018, and 2019–2025 (Table 1). This Subgroup analysis shows an increase in penicillin resistance from 1990 to 2013 (WPR: 44.6%; 95% CI: 38–51.4%) to 2014–2018 (WPR: 51.2%; 95% CI: 40.7–61.6%), followed by a partial decline in 2019–2025 (WPR: 47.1%; 95% CI: 39.3–55.1%). Although the trend Suggests a transient rise during 2014–2018 with a subsequent slight decrease, confidence intervals overlap, indicating no statistically significant change over time (*P* = 0.575). Overall, resistance rates remained relatively stable with modest fluctuations. Significantly, there was a difference in penicillin resistance rates between countries/continents, and AST (*P* < 0.001). Meta-regression confirmed that the penicillin resistance proportion decreased over time (*r*=−0.005, *P* = 0.44; Fig. 4). Among the 59 countries that reported penicillin resistance data, 31 (52.5%) reported that more than 40% of isolates were resistant (Fig. 3). Significantly, there was a difference in penicillin resistance rates between countries/continents (*P* < 0.001). Africa reports the highest prevalence of penicillin resistant bacteria resistance at a WPR of 79.3%. There was a significant difference in the AST methods used (*P* = 0.02). There was no significant difference between CLSI and EUCAST (*P* = 0.25) (Table 1).

### Ceftriaxone resistance

The determination of Susceptibility to ceftriaxone was carried out in a total of 201 studies, encompassing 102,448 isolates of NG. The WPR was found to be 0.7% (95% CI 0.6%−0.9%), although there was noticeable heterogeneity (I^2^ = 89.05%, *P* < 0.001) observed among the included articles (Table 1; Fig. 2). We found strong evidence of publication bias (Egger’s test, *P* < 0.001). This Subgroup analysis revealed an increase in the resistance rate when comparing the data from 1988 to 2013 (WPR 0.5%; 95% CI 0.3%−0.7%), 2014–2018 (WPR 0.7%; 95% CI 0.4%−1.2%), and 2019–2025 (WPR 1.1%; 95% CI 0.8%−1.6%) (Table 1). The proportion of ceftriaxone resistance isolates exhibited a significant variation over time (*P* = 0.010). Meta-regression confirmed that the ceftriaxone resistance proportion increased over time (*r* = 0.06, *P* = 0.419; Fig. 3).

Among the 64 countries that reported resistance data for ceftriaxone, 7 (28.3%) countries (such as Vietnam, Slovenia, Estonia, South Korea, Iran, Cambodia, and Switzerland) reported that more than 2% of specimens demonstrated ceftriaxone resistance (Fig. 4). Significantly, there was a difference in ceftriaxone resistance rates between continents (*P* < 0.001). Asia reports the highest prevalence of ceftriaxone resistant bacteria resistance at a WPR of 1.4%. In contrast, Africa, America, and Europe exhibited significantly Lower resistance rates of 0.8%, 0.3%, and 0.6%, respectively. In terms of individual countries within Asia, the ceftriaxone resistance rate of NG isolates was higher in Cambodia and Iran compared to other Asian countries (15.4% vs. 8.8%). There was a significant difference in the AST methods used (*P* = 0.02). There was no significant difference between CLSI and EUCAST (*P* = 0.25) (Table 1).

### Cefixime resistance

The Susceptibility to cefixime was assessed in a total of 92 studies, encompassing 72,193 isolates of NG. The WPR was estimated to be 2.5% with a 95% confidence interval (CI) ranging from 1.9 to 3.3%. Considerable heterogeneity (I^2^ = 96.13%) was observed among the included studies. Additionally, the Egger rank correlation test found a notable publication bias (*P* < 0.001). A subgroup analysis showed a statistically significant difference in cefixime resistance proportions over time (*P* < 0.001). The prevalence of cefixime resistance increased between the earliest and middle periods, rising from 0.9% (95% CI: 0.5–1.9) among 282 strains collected from 1988 to 2013 to 3.7% (95% CI: 2.4–5.7) among 665 strains collected from 2014 to 2018 — approximately a 4.1-fold increase. In the most recent period (2019–2025), resistance was 3.3% (95% CI: 2.2–4.8) among 1,112 strains; this represents a slight decline from 2014 to 2018 but remains Substantially higher than the 1988–2013 baseline (*P* ≤ 0.0001). Meta-regression confirmed that the cefixime resistance proportion increased over time (*r* = 0.07, *P* = 0.021; Fig. 3). Among the 39 countries that reported resistance data for cefixime, six of these countries (Bangladesh, Iran, Slovenia, China, Japan, and Italy) indicated that over 5% of isolates exhibited resistance (Fig. 4). Different continents had varying rates of resistance to cefixime, and this difference was statistically significant (*P* < 0.001). Specifically, Asia had a high resistance rate of 5.1%, while Africa, America, and Europe had significantly Lower resistance rates of 0.8%, 0.9%, and 2.4%, respectively. There is no significant difference between the AST methods and AST guidelines (*P* > 0.05).

###  Cefotaxime resistance

The resistance to cefotaxime was assessed in 59 reports that encompassed 47,233 NG isolates. The WPR was determined to be 1.5% (95% CI 1%−2.3%) with a considerable level of heterogeneity (I^2^ = 97.19%) observed among the included reports (Table 1; Fig. 2). Moreover, no substantial publication bias was identified (*P* < 0.001). As depicted in Table 1, there was a noteworthy difference in the proportion of cefixime resistance isolates during time (*P =* 0.035). The prevalence of cefotaxime resistance increased from 0.8% (95% CI 0.5–1.5) in 1988–2013 to 5.8% (95% CI 3.8–8.9) in 2014–2018. In 2019–2025 the pooled prevalence decreased to 1.5% (95% CI 0.8–2.6), representing roughly a two‑fold reduction compared with the 2014–2018 peak (Table 1). Meta-regression confirmed that the cefotaxime resistance proportion increased over time (*r* = 0.014, *P* = 0.114; Fig. 3).

Among the 31 countries that reported resistance data for cefotaxime, Switzerland, Cambodia South Korea, Israel, Australia, and China reported that more than 5% of the specimens exhibited resistance (Fig. 4). A substantial change in cefotaxime resistance rates was observed across continents (*P* < 0.001). In contrast to Asia, which exhibited the highest cefotaxime resistance rate (WPR: 5.4%; 95% CI: 3.2–8.9%), other continents showed Lower Levels of resistance. Africa had a WPR of 1.0% (0.2–4.3%), Europe 2.0% (1.3–3.0%), and the Americas the Lowest at 0.3% (0.2–0.5%). Oceania showed a resistance rate of 1.3% (0.0–33.7%), though based on limited data.

No substantial heterogeneity was observed in cefotaxime resistance rates by AST method (*P* = 0.857), indicating consistency across testing techniques. However, a significant disparity was observed in resistance interpretation according to the guidelines used (CLSI, EUCAST, WHO), with statistically different estimates across standards (*P* = 0.035) (Table 1).

## Discussion

This study was conducted to determine the global prevalence of penicillin and ESCs resistance among NG isolates. It is very important to always keep an eye on the resistance that antibiotics can develop in bacteria in order to treat patients better. The appearance and growth of antimicrobial resistance in NG isolates have attracted attention all around the world [1, 2]. Since 1992, the WHO Gonococcal AMR Surveillance Programme (WHO-GASP) has been documenting AMR in gonorrhea globally, with the aim of improving resistance-testing methods and developing sentinel surveillance of NG AMR [268].

According to our meta-analysis, the worldwide prevalence of ESCs resistance in NG over the past 30 Years has remained at or below 2.5%. The WHO recommends discontinuing routine antibiotic treatment and using alternative agents when the NG AMR rate exceeds the 5% threshold [268, 269]. ESCs have been the cornerstone of gonorrhea antibiotic treatment, with ceftriaxone being the last remaining option for empirical first-line treatment [4–6]. In our meta-analysis, the WPRs of penicillin, ceftriaxone, cefixime, and cefotaxime were reported as 47.3, 0.7%, 2.5%, and 1.5% respectively. However, there have been recent reports of gradual decline in susceptibility or resistance to ESCs globally, and the fitness of NG ESCs resistance remains unknown, which may result in increased treatment failures [4–6][1, 2]. In 2018, the WHO-GASP found that more than 5% of samples from 7 countries (10. 8%) were less responsive or resistant to certain antibiotics in WHO regions [1, 2]. In our study, out of the 71 countries that provided data on ESCs resistance, 13 countries (18. 3%) had ESCs resistance rates higher than 2%. The rates of ESC resistance in NG bacteria differ in different regions because of various factors. These factors include differences in how they control and prevent gonorrhea, how they use antibiotics responsibly, whether they implement response plans effectively, if they misuse antimicrobials, and variations in how they test for antibiotic Susceptibility. Another meta-analysis conducted in 2014 demonstrated a global cefixime resistance rate of 0.2%, which is lower than our findings (2.5%) [270]. In addition, the rate of cefixime resistance among NG isolates originating from Asia exhibited a significantly Lesser extent compared to those from other continents; a finding that contradicts the outcomes of our meta-analysis. In addition, the rate of cefixime resistance among NG isolates from Asia was reported as 2.6%, which is significantly Lower than rates observed in other continents. However, our 2025 meta-analysis reveals a Substantially higher pooled resistance rate of 5.1% in the same region, indicating an upward trend over the past decade and suggesting underestimation in earlier estimates.

Consistent with prior meta-analyses [202, 203], the worldwide prevalence rate of ceftriaxone resistance remains below the established threshold of 5% [271, 272]. Throughout the duration of our investigation, certain variations were observed, yet a noteworthy escalation in the proportion of isolates demonstrating resistance to ESCs was detected over time, corroborating findings from other studies [270, 272]. Our research shows that the number of isolates resistant to ceftriaxone has gone up from 1988 to 2013 to 2019–2025. In the earlier period, there were 174 resistant bacteria, but in the later period, there were 653 resistant bacteria. Moreover, the frequency of cefixime-resistant isolates has exhibited a more than three-fold rise over time. Notably, the prevalence of ceftriaxone, cefixime and cefotaxime resistance among isolates in Asia surpasses that of other continents. These results underscore the existence of diverse patterns of ESCs resistance across different geographical areas and time periods.

According to this issue, ceftriaxone is currently the final option utilized in several nations to combat gonorrhoeae. Consequently, the gradual escalation in the occurrence of NG isolates exhibiting reduced susceptibility or resistance to ceftriaxone is a cause for concern that necessitates ongoing monitoring and investigation. The rise in resistance emergence of NG strains to ESCs has been postulated to be linked to genetic alterations within the *penA* genes chromosomal region (which encodes the transpeptidase domain of the PBP2 protein), the *porB1b* gene (which encodes the porin B subunit), the *ponA* gene (which encodes the PBP1 protein), as well as the overexpression of MtrCDE membrane pump proteins [271, 273, 274]. Updated analysis reveals these high-risk genotypes are increasingly prevalent in East and Southeast Asia (e.g., China, Thailand, Vietnam), with growing reports in North America, Europe, and Australia, often linked to international transmission [275–281]. Their expanding geographic distribution underscores the need for enhanced molecular surveillance to guide treatment policies and prevent global spread.

The high global prevalence of penicillin resistance in NG, as demonstrated in this meta-analysis (WPR: 47.3%; 95% CI: 42.7–51.9%), is largely driven by well-established molecular mechanisms, including the acquisition of penicillinase (via *blaTEM* genes), alterations in penicillin-binding proteins (PBPs), and mutations in porin genes (*porB*) that reduce drug permeability. Additionally, efflux pump overexpression (e.g., *mtrR* mutations) contributes to decreased intracellular antibiotic accumulation, further enhancing resistance. These mechanisms—often present in combination—explain the widespread and persistent nature of penicillin resistance, rendering this agent clinically ineffective for empirical gonorrhea treatment. The observed geographic disparity—with Africa exhibiting the highest resistance rate (WPR: 79.3%)—can be attributed to a confluence of factors: unregulated antibiotic use, limited access to advanced diagnostics, incomplete treatment regimens, and high transmission rates in underserved populations. In contrast, lower resistance in the Americas and parts of Europe may reflect stronger antimicrobial stewardship, Surveillance systems, and greater access to alternative therapies Such as extended-spectrum cephalosporins. The finding that 52.5% of reporting countries (31/59) have resistance rates exceeding 40% underscores the global irrelevance of penicillin monotherapy. Although resistance fluctuated over time—peaking at 51.6% during 2014–2018 before declining slightly to 48.2% in 2019–2025—this trend was not statistically significant (*P* = 0.575), suggesting overall stability. This plateau may reflect saturation of resistance determinants in circulating strains, where further increases are constrained by fitness costs or widespread fixation of resistance genes (e.g., *blaTEM* plasmids now endemic in many regions). The lack of a significant decline over time (*r* = − 0.005, *P* = 0.44) further supports the idea that once acquired, resistance mechanisms are stably maintained in the population. Our finding of significant heterogeneity by AST method (*P* = 0.02) aligns with prior studies showing that MIC-based methods tend to yield more precise resistance estimates compared to disk diffusion, particularly for fastidious organisms like NG. However, the absence of a significant difference between CLSI and EUCAST guidelines (*P* = 0.25) suggests that interpretive criteria are less influential than technical methodology or regional epidemiology. These results are consistent with—and extend—the findings of the 2014 WHO global antimicrobial resistance surveillance report, which also documented high penicillin resistance worldwide. However, our updated analysis (2025) reveals that resistance remains entrenched three decades later, with no meaningful reversal despite global control efforts. This contrasts with trends in some high-income countries where targeted interventions have reduced gonorrhea incidence, but highlights the persistent challenge in resource-limited settings.

This study has several limitations. First, the majority of included studies originated from middle- and high-income countries, with limited data from low-income regions—particularly in Africa and parts of Asia—potentially biasing global estimates and limiting generalizability. Second, substantial heterogeneity (I² >89%) was observed, largely due to variability in AST methods and laboratory protocols, which may affect the comparability of resistance rates. Third, while significant temporal trends were detected, the predominance of cross-sectional data limits the ability to establish causal inferences or assess long-term dynamics.

Additionally, differences in resistance interpretation criteria (e.g., CLSI, EUCAST, national guidelines) may influence pooled estimates, despite no significant difference found between major guidelines. The analysis focused primarily on ESCs, potentially overlooking trends in resistance to other clinically relevant antibiotics (e.g., azithromycin, doxycycline). Finally, contextual factors such as antibiotic consumption, healthcare access, and infection control practices were not systematically captured, despite their known impact on resistance evolution. These gaps highlight the need for standardized, longitudinal, and globally representative surveillance systems.

## Conclusions

This meta-analysis found Low but increasing global resistance to ESCs, with a WPR of 0.7% for ceftriaxone, 2.5% for cefixime, and higher regional rates for cefotaxime—reaching 5.4% in Asia. Although overall resistance remains below 3%, a significant upward trend over time (*P* = 0.010 for ceftriaxone; *P* < 0.001 for cefixime) is concerning, particularly given the unknown biological fitness cost of resistance in NG. The widespread geographic distribution of resistant strains—especially in Asia—confirms global dissemination of less susceptible isolates. These findings highlight the failure of older antibiotics like penicillin and underscore the urgent need for prudent antibiotic use, enhanced contact tracing, and strengthened antimicrobial resistance surveillance to preserve the effectiveness of current first-line therapies.

## Data Availability

All the information in this review is written in the manuscript.

## Abbreviations

AMR: Antimicrobial Resistance
NG: *Neisseria gonorrhoeae*
ESCs: Extended-spectrum cephalosporins
AST: Antimicrobial susceptibility testing
STI: Sexually transmitted infection
WPR: Weighted pooled resistance rate
PRISMA: Preferred Reporting Items for Systematic Reviews and Meta Analyses
CLSI: Clinical and Laboratory Standards Institute
EUCAST: European Committee on Antimicrobial Susceptibility Testing
CI: Confidence interval
WHO-GASP: WHO Gonococcal AMR Surveillance Programme
NAAT: Nucleic acid amplification testing
